# Age and sex differences in vasovagal syncope: triggers, clinical presentation, prodromal symptoms, and head-up tilt test results

**DOI:** 10.1093/ehjopen/oeaf061

**Published:** 2025-06-11

**Authors:** Mohammadreza Babaei, Masih Tajdini, Ali Bozorgi, Saeed Sadeghian, Morvarid Taebi, Hamed Tavolinejad, Mehrdad Mahalleh, Homa Taheri, Florian Rader, Jeffrey R Boris, Artur Fedorowski

**Affiliations:** Cardiovascular Diseases Research Institute, Tehran Heart Center, Tehran University of Medical Sciences, North Kargar Street, Tehran 1411713138, Iran; Cardiovascular Diseases Research Institute, Tehran Heart Center, Tehran University of Medical Sciences, North Kargar Street, Tehran 1411713138, Iran; Division of Cardiology, Johns Hopkins Hospital, The Johns Hopkins University School of Medicine, Baltimore, MD 21205, USA; Cardiovascular Diseases Research Institute, Tehran Heart Center, Tehran University of Medical Sciences, North Kargar Street, Tehran 1411713138, Iran; Cardiovascular Diseases Research Institute, Tehran Heart Center, Tehran University of Medical Sciences, North Kargar Street, Tehran 1411713138, Iran; Cardiovascular Diseases Research Institute, Tehran Heart Center, Tehran University of Medical Sciences, North Kargar Street, Tehran 1411713138, Iran; Cardiovascular Diseases Research Institute, Tehran Heart Center, Tehran University of Medical Sciences, North Kargar Street, Tehran 1411713138, Iran; Division of Cardiovascular Medicine, Perelman School of Medicine, 3400 Civic Center Blvd, Philadelphia, PA 19104, USA; Cardiovascular Diseases Research Institute, Tehran Heart Center, Tehran University of Medical Sciences, North Kargar Street, Tehran 1411713138, Iran; Department of Cardiology, Cedars-Sinai Medical Center, 8700 Beverly Blvd, Los Angeles, CA 90048, USA; Department of Cardiology, Cedars-Sinai Medical Center, 8700 Beverly Blvd, Los Angeles, CA 90048, USA; Jeffrey R. Boris, MD, LLC, P.O. Box 16 Moylan, PA 19065, USA; Department of Cardiology, Karolinska University Hospital, 171 76 Stockholm, Sweden; Department of Medicine, Karolinska Institute, Stockholm 17177, Sweden

**Keywords:** Vasovagal syncope, Prodrome, Head-up tilt test, Trigger, Haemodynamic

## Abstract

**Aims:**

Previous studies show inconsistencies in vasovagal syncope (VVS) symptoms and haemodynamic responses across age and sex groups, with limited evaluation of tilt test results. This study comprehensively examines differences in triggers, prodromal and syncopal symptoms, and head-up tilt test (HUTT) responses among VVS patients by age and sex providing new insights.

**Methods and results:**

We analysed data from Syncope Unit of Tehran Heart Center, including adults (≥18 years) with suspected VVS diagnosis based on clinical history and physical exams according to syncope guidelines, to explore sex- and age-specific clinical features and HUTT outcomes. The study included 1914 VVS patients (mean age: 46.6 ± 17.8; 51.3% male). Males were more likely to experience first-time syncope (31.6% vs. 19.8%, *P* < 0.001), whereas females had more recurrent episodes (37.5% vs. 31.2%, *P* < 0.01) and reported more identifiable triggers. During the HUTT passive phase, females exhibited a greater diastolic blood pressure drop [49.5 ± 12.2 vs. 34.4 ± 17.2, *P* = 0.012], while in the active phase, they experienced a more pronounced heart rate reduction 39.7 ± 26.9 vs. 30.2 ± 23.3, *P* < 0.001. Cardioinhibitory syncope was more prevalent in younger patients, with over two-thirds of cases occurring in individuals under 50 years old, and its frequency declined with age. In contrast, vasodepressor syncope peaked in the 51–70 age group. Agreement between spontaneous and HUTT-induced syncope was low (κ = 0.06–0.32).

**Conclusion:**

Age and sex shape VVS presentation, triggers, and haemodynamic response, emphasizing the need for demographic considerations in management and the limitations of HUTT.

## Graphical Abstract

**Graphical Abstract:**
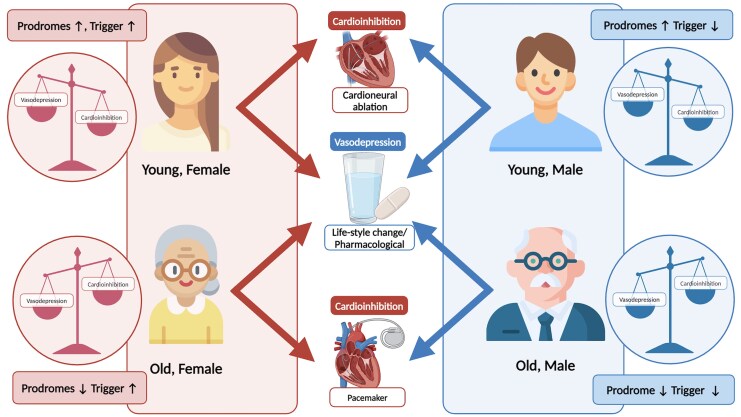

## Introduction

Vasovagal syncope (VVS) is a transient loss of consciousness resulting from a sudden drop in the heart rate and blood pressure, leading to reduced cerebral blood flow and a loss of postural tone.^1^ Vasovagal syncope affects millions of people worldwide, with a lifetime incidence of ∼40%.^2^ Despite its prevalence, understanding its underlying mechanisms and clinical presentation remains limited.^3,4^

Previous studies suggest that sex and age significantly affect VVS incidence, triggers, outcomes, and responses to the head-up tilt test (HUTT).^5–10^ For instance, females often report more prodromal symptoms than males, and younger individuals may experience VVS more frequently from psychological triggers.^11^ Recognizing these symptoms and triggers could reduce injuries and morbidity.^12^ Additionally, studies indicate that HUTT responses vary by age and sex, with older patients more likely to show a vasodepressor response rather than a cardioinhibitory response.^6^ Although HUTT has been widely used as a diagnostic test in suspected cases of VVS, its results may not always align with clinical diagnoses of VVS based on patient’s history and typical syncope presentation. However, according to recent studies, HUTT’s relevance for mechanism-based therapy in syncope related with autonomic dysfunction, previously termed ‘non-cardiac syncope’ has been strengthened.^13,14^

This study aims to enhance the understanding of VVS by analysing sex- and age-specific differences across all aspects of the condition, from symptomatology to diagnosis, utilizing the largest known sample size to comprehensively assess these variations. In addition to thoroughly examining haemodynamic changes and response types during the HUTT, we compared symptoms during spontaneous and HUTT-induced syncope to determine whether HUTT results can reliably predict patients’ real-world prodrome symptoms for clinical guidance.

## Methods

**Figure 1:**
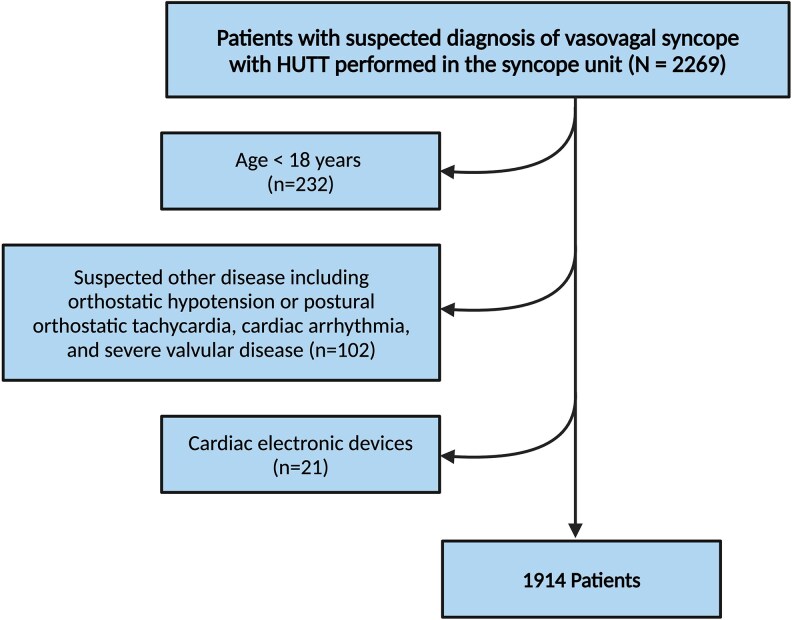
Study flowchart. HUTT, head-up tilt test.

The present investigation is a registry-based study exploring the dataset from Syncope Unit of Tehran Heart Center. We included all adult patients aged 18 years or older, referred to the tertiary syncope unit between 2017 and 2022, who had a suspicion of VVS based on the primary care physician’s assessment and who consequently underwent HUTT. The suspicion of VVS was based on the patient’s clinical history, as provided by patients, families, and witnesses of the syncopal event, along with physical examination, active standing test, and 12-lead electrocardiogram according to the current syncope guidelines.^1,15^ The physician had the option to conduct tilt test to record whether the case appeared to be typical VVS based on the clinical history. Exclusion criteria consisted of evidence of orthostatic hypotension or postural orthostatic tachycardia, defined by a decrease in blood pressure ≥ 20/10 mmHg or an increase in heart rate ≥ 30 b.p.m. during active standing test, cardiac arrhythmia, severe valvular disease or presence of electronic cardiac device such as permanent pacemaker or implantable cardioverter defibrillator. *Figure 1* summarizes the study’s patient selection process.

To further understand the sex- and age-specific characteristics of VVS, demographic data, including age, sex, weight, height, and past medical history, such as diabetes mellitus, hypertension, and anaemia, were selected based on their potential impact on cardiovascular function and their prevalence in the study population. Clinical features present at the time of syncope were also recorded and assessed during the study. Initial workup for each patient included an electrocardiogram, physical examination, and in selected cases, echocardiography. Complementary interventions, such as additional testing, were determined based on the clinical judgment of the syncope unit physician. Data collection involved patient interviews, physical examinations, and information provided by witnesses of the syncopal event, which were recorded in a questionnaire by nurse investigators. The recorded clinical features included the number of episodes experienced within the past year and throughout the patient’s lifetime, history of emergency department visits, triggers for VVS, and prodromal symptoms. Triggers for VVS were defined as specific stimuli that can precipitate a syncopal episode, such as crowded or warm environments, exposure to hot water (e.g. shower), sight of blood, emotional stress, pain, and medical or dental procedures. Prodromal symptoms, which are warning signs that occur before a syncopal episode, were also documented and included light-headedness, blurred vision, diaphoresis (excessive sweating), nausea, palpitation (rapid or irregular heartbeat), heat feeling, chest pain, abdominal discomfort, flushing, and aura (perceptual disturbances like flashing lights, blind spots, tingling or numbness, or other sensory experiences like unusual smells and tastes). Additionally, signs and symptoms during the syncopal event (e.g. seizure-like movements, abnormal gaze, urinary incontinence, and tongue bite), as well as the presence of post-syncopal symptoms (e.g. retrograde amnesia, headache, drowsiness, chest pain, and palpitation), were documented.

The HUTT was conducted using an electrically controlled tilt table, following the Italian protocol.^16^ Before commencing the test, patients underwent a stabilization phase of 5–10 min of rest in the supine position. Patients were required to discontinue all medications that could potentially affect the HUTT results, including all classes of antihypertensive medications and vasodilators, for an appropriate period before the test (i.e. five half-lives), as per standard protocol. The interpretation of the HUTT result and the type of response were determined according to the Vasovagal Syncope International Study (VASIS) criteria.^17^ During HUTT, symptoms were captured in real-time by the attending physician, who documented the presence/absence of symptoms. A positive HUTT result was defined only if syncope occurred during the test, while pre-syncope or a decrease in blood pressure without complete loss of consciousness (LOC) was not considered a positive outcome. The determination of complete LOC was made by the attending physician conducting the tilt test.

### Ethical considerations

The study was conducted in accordance with the Declaration of Helsinki and received ethical approval from the institutional committee for ethics in biomedical research (ID: IR.TUMS.MEDICINE.REC.1400.1485). All participants provided written informed consent for the use of their anonymous information in the registry.

### Statistical analysis

All analyses were conducted using R (version 4.2, R Foundation for Statistical Computing). The Kolmogorov–Smirnov test assessed normality for continuous variables, which are presented as mean ± standard deviation (compared using independent samples *t*-tests) or median (25th–75th percentiles; compared using Mann–Whitney *U* tests) for skewed data. Categorical variables are reported as frequencies (percentages) and analysed with χ^2^ or Fisher’s exact tests. To evaluate whether HUTT-induced prodromes predict real-world symptoms, we calculated Cohen’s kappa (κ) with 95% confidence intervals (CIs) for individual symptom agreement (<0.20: poor; 0.21–0.40: fair; 0.41–0.60: moderate; 0.61–0.80: good; >0.80: excellent) and used McNemar’s test to detect systematic differences in symptom reporting. A two-sided *P*-value of <0.05 defined statistical significance.

## Results

From the 2269 patients in the Tehran Heart Center syncope registry who underwent HUTT, 232 were excluded for being under 18 years old. Additionally, 102 cases with suspected alternative causes of transient loss of consciousness or an uncertain VVS diagnosis, as well as 21 patients with cardiac implantable electronic devices, were also excluded. This resulted in a final study population of 1914 patients with a mean age of 46.6 ± 17.8.

### Patient characteristics

Of the 1914 patients, 932 were female (48.7%) and 982 were male (51.3%). Male participants had a significantly higher mean age of 48.7 ± 19.0 years compared to females (44.0 ± 16.1, *P* < 0.001) at the time of performing HUTT. The age distribution of the study population, stratified by decade, is shown in Supplementary material online, *Figure S1*. The overall prevalence of diabetes mellitus, hypertension, and anaemia was 8.4%, 22.4%, and 17.2%, respectively. These conditions were similarly prevalent in both sexes, except for anaemia, which was significantly more common in females (24.2% vs. 10.5%, *P* < 0.001) (*Table 1*). One hundred and ten patients had visited the emergency department (ED) for a syncopal episode. There was a non-significant trend of more ED visits among males compared to females (7% vs. 5%, *P* = 0.092). However, these visits significantly increased with age, reaching 15% in patients older than 70 (*Table 1*). Additionally, 453 patients (25.9) experienced syncope for the first time in their lives. Males presented more frequently with first-time syncope (32% vs. 20%, *P* < 0.001) and had fewer recurrences compared to female participants (68% vs. 80%, *P* < 0.001) (*Table 1*). Comparing the number of syncopal episodes and syncope-related injuries across different age groups showed that recurrent episodes were more likely to occur in younger patients, although there was no significant difference in the rate of injury (*Table 1*).

**Table 1:** Characteristics and differences of the total study population across age groups

		Sex group			Age group (year)				
	Overall (*n* = 1914)	Female (*n* = 932)	Male (*n* = 982)	*P*-value	18–30 (*n* = 446)	31–50 (*n* = 660)	51–70 (*n* = 601)	>70 (*n* = 207)	*P*-value
Age, years	46.4 ± 17.8	44.0 ± 16.1	48.7 ± 19.0	<0.001	–	–	–	–	–
Female sex (%)	–	–	–	–	220 (49.3)	387 (58.6)	260 (43.3)	65 (31.4)	<0.001
Body mass index, kg/m^2^	26.1 ± 4.7	26.4 ± 5.3	25.9 ± 4.1	0.007	23.6 ± 4.7	26.7 ± 4.8	27.5 ± 4.2	25.8 ± 3.7	<0.001
Diabetes mellitus (%)	161 (8.4)	68 (7.3)	93 (9.5)	0.103	6 (1.3)	26 (3.9)	85 (14.1)	44 (21.3)	<0.001
Hypertension (%)	428 (22.4)	181 (19.4)	247 (25.2)	0.003	21 (4.7)	92 (13.9)	215 (35.8)	100 (48.3)	<0.001
Anaemia (%)	329 (17.2)	226 (24.2)	103 (10.5)	<0.001	78 (17.5)	124 (18.8)	95 (15.8)	32 (15.5)	0.483
Syncopes in last year (%)				0.010					<0.001
1	803 (45.9)	355 (41.9)	448 (49.6)		157 (38.2)	272 (44.6)	273 (50.6)	101 (52.9)	
2	348 (19.9)	175 (20.6)	173 (19.2)		86 (20.9)	116 (19.0)	106 (19.7)	40 (20.9)	
3	176 (10.1)	92 (10.8)	84 (9.3)		39 (9.5)	61 (10.0)	54 (10.0)	22 (11.5)	
≥4	424 (24.2)	226 (26.7)	198 (21.9)		129 (31.4)	161 (26.4)	106 (19.7)	28 (14.7)	
Missing	163 (100)	84 (51.5)	79 (48.5)		35 (21.5)	50 (30.7)	62 (38.0)	16 (9.8)	
Lifetime syncopes (%)				<0.001					<0.001
1	453 (25.9)	168 (19.8)	285 (31.6)		74 (18.0)	140 (23.0)	162 (30.1)	77 (40.7)	
2	348 (19.9)	173 (20.4)	175 (19.4)		70 (17.0)	118 (19.3)	122 (22.6)	38 (20.1)	
3	244 (14.0)	116 (13.7)	128 (14.2)		49 (11.9)	96 (15.7)	70 (13.0)	29 (15.3)	
≥4	704 (40.3)	391 (46.1)	313 (34.7)		218 (53.0)	256 (42.0)	185 (34.3)	45 (23.8)	
Missing	165 (100)	84 (50.9)	81 (49.1)		35 (21.2)	50 (30.3)	62 (37.6)	18 (10.9)	
History of syncope associated injury (%)	676 (35.3)	346 (37.1)	330 (33.6)	0.108	155 (34.8)	219 (33.2)	229 (38.1)	73 (35.3)	0.711
ED visit for syncope (%)	110 (5.7)	45 (4.8)	65 (6.6)	0.092	11 (2.5)	21 (3.2)	47 (7.8)	31 (15.0)	<0.001
HUTT results (%)				0.328					0.018
Negative	1150 (60.1)	549 (58.9)	601 (61.2)		241 (54.0)	412 (62.4)	376 (62.6)	121 (58.5)	
Positive	764 (39.9)	383 (41.1)	381 (38.8)		205 (46.0)	248 (37.6)	225 (37.4)	86 (41.5)	

Categorical variables are presented as numbers (percentages) and continuous variables as mean ± standard deviation.

ED, emergency department; HUTT, head-up tilt test.

### Triggers and symptoms

Female patients were more likely to have an identifiable and avoidable trigger for VVS (47% vs. 34%, *P* < 0.001). The most common trigger for both sexes was being in a crowded or warm environment (21% vs. 11%, *P* < 0.001). Other triggers included exposure to hot water (14% vs. 10%, *P* = 0.002), emotional stress (10% vs. 7%, *P* = 0.014), pain (11% vs. 6%, *P* < 0.001), sight of blood (9% for both sexes, *P* = 0.91), and undergoing medical or dental procedures (7% vs. 3%, *P* < 0.001). All triggers, except for the sight of blood, were significantly more frequent in females (*Table 2*). Comparing these triggers across different age groups showed that older patients had a lower probability of having specific triggers before VVS (23%), whereas about half of the patients aged 18–50 experienced syncope following these triggers (*Table 2*). A detailed description of triggers in patients with positive HUTT is presented in Supplementary material online, *Table S1*.

**Table 2:** Syncope triggers based on sex and age groups

Total population		Sex group			Age group (years)				
	Overall (*n* = 1914)	Female (*n* = 932)	Male (*n* = 982)	*P*-value	18–30 (*n* = 446)	31–50 (*n* = 660)	51–70 (*n* = 601)	>70 (*n* = 207)	*P*-value
Presence of any triggers (%)	769 (40.2)	435 (46.7)	334 (34.0)	<0.001	237 (53.1)	287 (43.5)	197 (32.8)	48 (23.2)	<0.001
Crowded/warm environment	305 (15.9)	195 (20.9)	110 (11.2)	<0.001	109 (24.4)	110 (16.7)	77 (12.8)	9 (4.3)	<0.001
Exposure to hot water	226 (11.8)	132 (14.2)	94 (9.6)	0.002	79 (17.7)	86 (13.0)	48 (8.0)	13 (6.3)	<0.001
Sight of blood	175 (9.1)	84 (9.0)	91 (9.3)	0.910	71 (15.9)	61 (9.2)	35 (5.8)	8 (3.9)	<0.001
Emotional stress	167 (8.7)	97 (10.4)	70 (7.1)	0.014	49 (11.0)	72 (10.9)	41 (6.8)	5 (2.4)	<0.001
Pain	158 (8.3)	103 (11.1)	55 (5.6)	<0.001	56 (12.6)	64 (9.7)	37 (6.2)	1 (0.5)	<0.001
Medical/dental procedures	93 (4.9)	65 (7.0)	28 (2.9)	<0.001	23 (5.2)	41 (6.2)	28 (4.7)	1 (0.5)	0.010

Data are presented as numbers (percentages). Note that percentages sum to >100% as patients could report multiple triggers.

**Figure 2:**
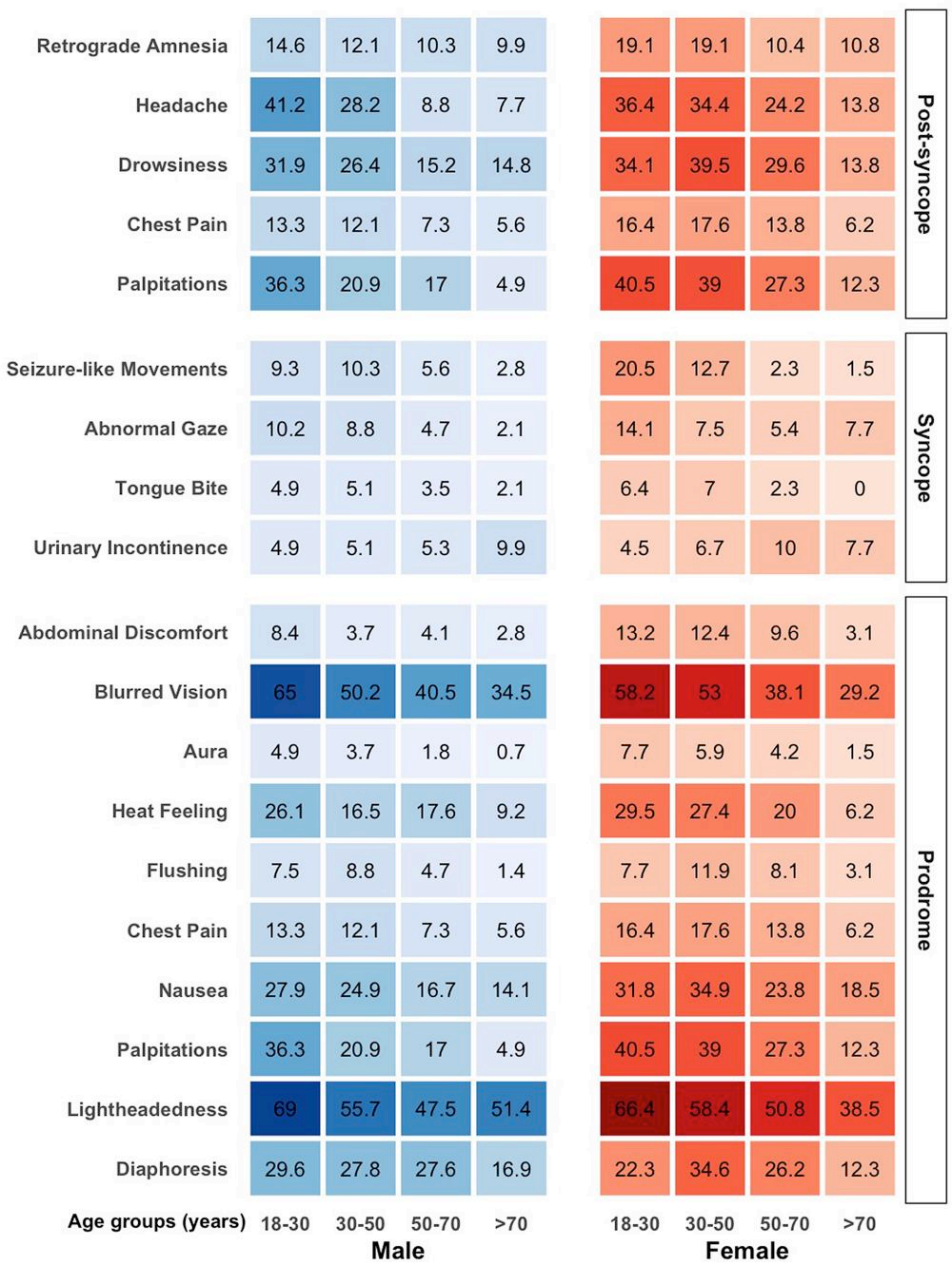
Peri-syncopal symptoms based on sex and age groups. The data are presented as percentages, with the intensity of shading reflecting the magnitude of the percentage values. Columns representing the male and female population are distinguished by labelling, and each group is divided into four columns based on age categories. Each row corresponds to a specific peri-syncopal symptom. For additional details, please refer to Supplementary material online, *Tables S2*–*S4*.

More than 80% of the patients experienced prodromes before VVS, with a marginally higher prevalence in females (84.2% vs. 80.4%) (see Supplementary material online, *Table S2*). However, comparisons across age groups showed that the likelihood of prodromal symptoms decreased significantly with age (*P* < 0.001). The most common prodromes reported were light-headedness (∼55%), and blurred vision (∼48%). Other prodromes, ranked by prevalence, included palpitations, nausea, diaphoresis, a sensation of heat, chest pain, abdominal discomfort, flushing, and aura. Except for light-headedness, blurred vision, and diaphoresis, all symptoms were significantly more frequently reported by females. As mentioned earlier, all prodromes were substantially more common in younger patients (*Figure 2*, Supplementary material online, *Table S2*).

Regarding syncopal symptoms, younger patients were more likely to present with atypical features such as seizure-like movements, tongue biting, or abnormal gaze at the time of syncope (*Figure 2*). Conversely, there was a non-significant trend for older patients to experience urinary incontinence during VVS. Notably, females, particularly at younger ages, were more prone to displaying seizure-like movements during syncope (20.5% in females between 18 and 30 years). Furthermore, post-syncopal symptoms were more prevalent among females, encompassing palpitations (23% vs. 15%, *P* < 0.001), drowsiness (34% vs. 22%, *P* < 0.001), headache (31% vs. 22%, *P* < 0.001), and chest pain (13% vs. 9%, *P* < 0.001). A comprehensive overview of all syncopal symptoms is presented in Supplementary material online, *Tables S3* and *S4*.

### Head-up tilt test findings

Of the 1914 patients, 764 (40%) had a positive HUTT result, with no significant difference between males and females (*P* = 0.3). The baseline characteristics of HUTT-positive patients based on age and sex are presented in Supplementary material online, *Table S5*. Comparing HUTT results across different age groups showed that patients aged 31–70 years had a lower probability of a positive HUTT compared to those under 30 and over 70 years (37.5% vs. 44.6%) (*Table 1*). More than two-thirds of the positive tests occurred during the active phase of HUTT (see Supplementary material online, *Table S6*).

**Figure 3:**
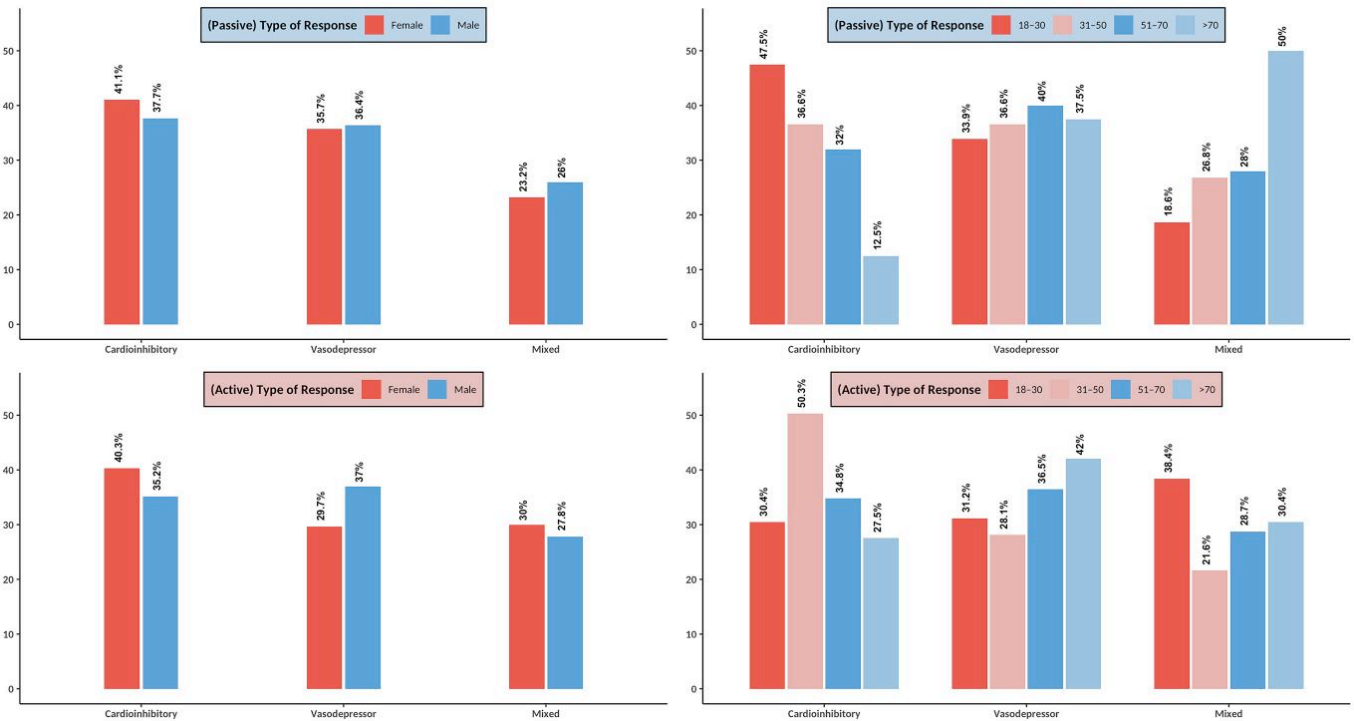
Response type in HUTT-positive patients based on sex and age groups. The data are presented as percentages, with rows representing the type of HUTT response (either passive or active test). Legends indicate whether results pertain to passive or active tests, and columns categorize the data based on sex and age. Abbreviation: HUTT, head-up tilt table.

**Figure 4:**
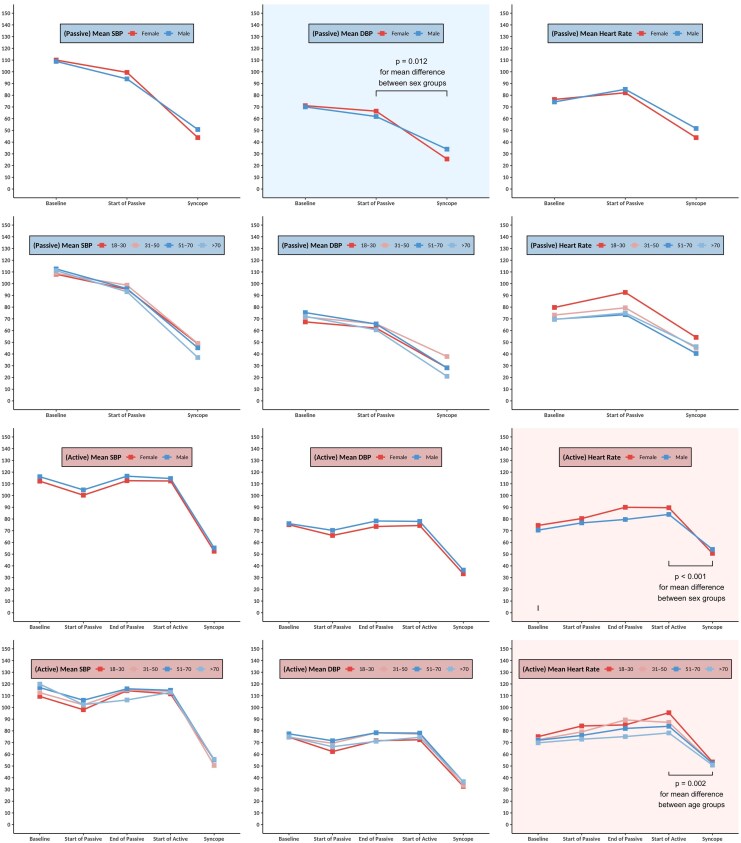
Haemodynamic changes during HUTT in patients with positive test. The data are presented as numerical values (mean), with columns representing systolic blood pressure, diastolic blood pressure, and heart rate, while rows categorize the data based on age and sex. Legends indicate whether results pertain to passive or active tests. Significant differences are denoted by a shaded background, with corresponding *P*-values displayed within the graphs. Abbreviation: HUTT, head-up tilt table.

Among the patients with positive HUTT, the distribution of syncope types varied between males and females. Although, there were no significant sex differences in the frequency of mixed, cardioinhibitory, or vasodepressor syncope (*Figure 3*). During the passive phase, females experienced a significantly greater mean diastolic blood pressure (DBP) reduction compared to males (49.5 ± 12.2 mmHg vs. 34.4 ± 17.2 mmHg, *P* = 0.012). Additionally, during the active phase, females exhibited a greater mean heart rate (HR) reduction than males (39.7 ± 26.9 b.p.m. vs. 30.2 ± 23.3 b.p.m., *P* < 0.001) (*Figure 4*, Supplementary material online, *Table S6*). Age-dependent differences in response to the active phase of the HUTT were noted in all subtypes of syncope (*Figure 3*). During the passive phase, cardioinhibitory syncope was found to be more prevalent among younger patients (47.5%), and progressively declined with age, reaching ∼12.5% in those older than 70 years (*Figure 3*, Supplementary material online, *Figure S2*, and Supplementary material online, *Table S6*). Also, there was a non-significant trend for greater reductions in both mean systolic and diastolic blood pressure with age, but these differences were not statistically significant (*Figure 4*). The overall trend again suggests that older patients may experience greater vasodepression, while younger individuals exhibit stronger cardioinhibitory reflexes.

In the active phase, cardioinhibitory syncope peaked in the 31–50 age group (50%) and declined in older patients (34/8% and 27.5% in 51–70 and >70 years old, respectively). Moreover, the prevalence of vasodepressor responses progressively increased with advancing age (*Figure 3*, Supplementary material online, *Table S6*). Significant differences in the haemodynamic responses were also apparent with age. Mean heart rate reduction during the active phase was highest in younger patients (42.6 ± 26.3 b.p.m. in the 18–30 age group) and progressively declined with age (28.2 ± 21.9 b.p.m. in those >70 years, *P* = 0.002). Haemodynamic changes in patients with negative HUTT results are presented in Supplementary material online, *Figure S3*.

### Head-up tilt test vs. spontaneous syncope prodromes

**Figure 5:**
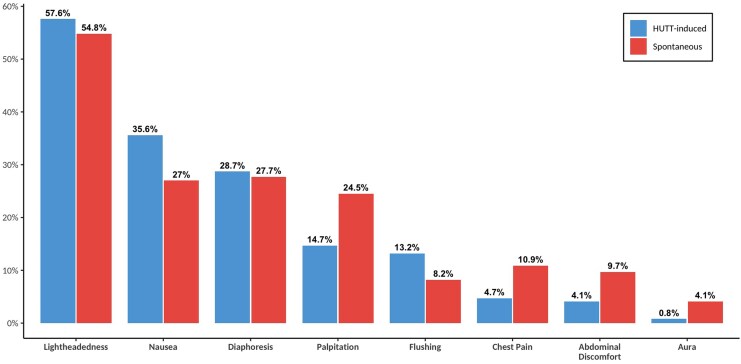
Spontaneous vs. HUTT-induced prodromal symptoms. HUTT, head-up tilt test; data are presented as percentages. Each bar represents a specific prodromal symptom common to both spontaneous and HUTT-induced syncope (based on data availability).

The most common prodromes experienced by patients with a positive HUTT were light-headedness (57.6%), nausea (35.6%), weakness (33.2%), and diaphoresis (28.7%) with palpitations (14.7%) the only symptom significantly more common in females (18% vs. 11%, *P* = 0.01). Comparing these symptoms across different age groups showed that patients older than 70 years were less likely to experience nausea and palpitations during HUTT, while other symptoms were comparable across age groups (see Supplementary material online, *Table S7*). Mutual prodromal symptoms between spontaneous and HUT-induced syncope were assessed for agreement (*Figure 5*). McNemar test revealed significant differences for several symptoms, including palpitation (χ² = 30.59, *P* < 0.001), nausea (χ² = 16.13, *P* < 0.001), chest pain (χ² = 24.32, *P* < 0.001), abdominal discomfort (χ² = 22.33, *P* < 0.001), flushing (χ² = 10.87, *P* < 0.001), and aura (χ² = 19.86, *P* < 0.001), indicating discrepancies in the occurrence of these symptoms. Cohen’s Kappa values ranged from 0.06 to 0.32, signifying slight to fair agreement. Specifically, diaphoresis (κ = 0.32), palpitation (κ = 0.27), and light-headedness (κ = 0.25) demonstrated fair agreement, whereas nausea, aura, chest pain, abdominal discomfort, and flushing exhibited slight agreement (*Table 3*). Overall, these results suggest a low level of agreement between symptoms. The agreement results were consistent between both sexes (see Supplementary material online, *Table S8*).

**Table 3:** Agreement between prodromal symptoms of spontaneous syncope and HUT-induced syncope

	McNemar’s test		Cohen’s kappa		
HUTT positive	χ^2^	*P*-value	*k*	95% CI	*P*-value
Diaphoresis	0.17	0.678	0.32	0.25–0.40	<0.001
Palpitation	30.59	<0.001	0.27	0.17–0.36	<0.001
Light-headedness	1.41	0.234	0.25	0.19–0.32	<0.001
Nausea	16.13	<0.001	0.21	0.13–0.29	<0.001
Chest pain	24.32	<0.001	0.22	0.06–0.37	0.006
Abdominal discomfort	22.33	<0.001	0.20	0.04–0.37	0.015
Flushing	10.87	<0.001	0.14	0.01–0.28	0.025
Aura	19.86	<0.001	0.21	−0.08–0.49	0.101
No symptom	0.82	0.365	0.06	−0.07–0.16	0.213

Kappa interpretation, ‘<0’: no agreement; ‘0–0.2’: slight agreement; ‘0.2–0.4’: fair agreement; ‘0.4–0.6’: moderate agreement; ‘0.6–0.8’: substantial agreement; ‘0.8–1.0’: almost perfect agreement.

CI, confidence interval; GI, gastrointestinal.

## Discussion

In this registry-based study, we analysed the demographics, clinical characteristics, and HUTT results of patients with VVS. Our findings suggest that gender and age significantly influence the presentation and characteristics of VVS, which can inform diagnostic and therapeutic approaches. Furthermore, our study revealed that male patients with VVS were older and more likely to present with first-time syncope compared to female patients with recurrent syncope more frequently seen among these patients. However, there was no significant difference in positive HUTT results between males and females. Older patients were less likely to present with specific triggers and prodromes before syncope but experienced a higher incidence of urinary incontinence during syncopal episodes. Additionally, the cardioinhibitory response was less common among elderly patients. These findings suggest that a more nuanced approach to VVS management is needed, one that incorporates both age-specific and sex-specific considerations for each individual to achieve more effective care.

Although syncope caused by vasovagal reflex is most prevalent and constitutes ∼50–60% of all initially unexplained cases,^18^ it should be emphasized that only around 2–3% of the whole population seek medical attention due to syncopal episodes.^19^ Beyond prevalence, complications associated with VVS, including anxiety, depression, and injury from falls (ranging from minor abrasions to serious fractures or head trauma), significantly impact the quality of life.^20^ These complications, besides clinical presentation, prodromal symptoms, and triggers, are suggested to be different in different sexes. It is known that sex differences in autonomic function may play a role in the pathophysiology of VVS, with females often exhibiting lower baseline blood pressure and potentially altered vagal tone.^10,21^ Additionally, variations in blood pressure regulation, iron deficiency, and hormonal changes during the menstrual cycle may increase symptom variability in females, with light-headedness, for example, fluctuating throughout the cycle.^22–24^ In contrast, male patients with VVS exhibit fewer prodromal symptoms and have fewer identifiable and avoidable triggers, which may complicate both diagnosis and treatment. The absence of typical features may make the diagnosis of VVS among males more challenging and the implementation of therapies more difficult.

Our study found that females, particularly younger ones, were more susceptible than males to experiencing VVS triggered by specific stimuli. Crowded or warm environments were the most common triggers, affecting 21% of females compared to 11% of males. Other triggers, including exposure to hot water, emotional stress, pain, and medical or dental procedures, were also more frequently reported by females, except for the sight of blood (*Table 2*). A study by Tajdini *et al*.^12^ showed that females are more susceptible to severe injuries from VVS while experiencing prodromal symptoms can be protective by enabling patients to take preventive actions like sitting or lying down. In the current study, approximately one-third of participants reported a history of injury from VVS. A straightforward approach to reducing the risk of VVS-related injuries involves educating patients on avoiding known triggers and recognizing prodromal symptoms to enable life-saving actions, such as using counter-pressure manoeuvres.^25^ Thus, categorizing syncope triggers and prodromes between different ages and sexes can result in a more complete ascertainment of history, easier diagnosis, and patient-centric management. In addition to these preventive measures, the categorization of haemodynamic responses to VVS has led to targeted treatment options based on patient phenotype. For instance, while hypotensive-predominant VVS often responds to lifestyle modifications or pharmacotherapy, cardioinhibitory phenotypes may require targeted interventions.^1,26^ These interventions include cardioneuroablation for younger patients to avoid lifelong pacemaker dependence, while pacemakers may be preferable for older individuals due to reduced autonomic adaptability and higher comorbidity risks.^27,28^ This study provides valuable insights into the distribution of VVS phenotypes according to demographic parameters, which can guide healthcare providers in selecting age- and phenotype-specific interventions.

Consistent with findings from the Prevention of Syncope Trial (POST),^10^ females in our study reported more prodromal symptoms, with light-headedness and blurred vision being the most common across both sexes. However, symptoms such as nausea, palpitations, flushing, feeling hot, abdominal discomfort, and aura were significantly more prevalent in females. Additionally, a higher proportion of females exhibited seizure-like movements during VVS episodes, a phenomenon noted in other studies^10,29^ that may increase the risk of misdiagnosis as epilepsy. While females in our study reported more post-syncopal symptoms—such as palpitations, drowsiness, headache, and chest pain—these symptoms did not translate to a higher incidence of trauma or emergency visits compared to males.^12^ This greater prevalence of post-syncopal symptoms in females may suggest a more pronounced reduction in cerebral blood flow during episodes (*Figure 2*).

In contrast, older patients with VVS faced unique challenges in diagnosis and treatment due to fewer identifiable triggers and prodromal symptoms, possibly stemming from reduced autonomic responsiveness and a diminished capacity to perceive symptoms.^9,30^ This decreased awareness, combined with a rapid onset of syncope, limits their ability to take preventive actions, such as adjusting body position or using counter-pressure manoeuvres. Older adults also reported fewer post-syncopal symptoms like drowsiness, headache, and retrograde amnesia, yet they visited the ED more frequently. This increased use of emergency services suggests that older adults with VVS may contribute to higher healthcare costs, as their VVS episodes are more likely to require acute medical attention

The HUTT yielded positive results in 40% of patients in our study, which is a lower rate than reported in prior studies.^31,32^ This discrepancy may stem from our protocol’s stricter criteria, as we classified the test as positive only if syncope occurred, aiming to improve diagnostic specificity. No significant sex-based differences were observed in positive HUTT results, although previous studies have shown a higher positivity rate among females.^32^ Age, however, appeared to influence the type of response as older patients exhibited a lower rate of cardioinhibitory responses, consistent with findings that autonomic control shifts with age.^6,9^ Previous studies have found that age affects the circulatory control of the autonomic system, as with increasing age, the response to orthostatic challenge relies more on the increase in peripheral resistance, whereas in younger adults, this response is regulated predominantly by an increase in heart rate.^30,33^

Our results show major differences in haemodynamic responses during HUTT according to sex and age. Although there was no difference in the overall distribution of syncope subtypes between males and females, females developed a larger diastolic blood pressure drop in the passive phase and a more profound heart rate drop in the active phase, suggesting heightened autonomic sensitivity. Age-related variations were also evident, with cardioinhibitory syncope being more prevalent in younger patients and declining with age, while vasodepressor syncope peaked in the 51–70 age group. These trends align with the known age-related shifts in autonomic function where younger individuals demonstrate stronger vagal responses, whereas older patients exhibit increased vasodepression.^9^ These findings emphasize that younger patients with predominant cardioinhibition may benefit more from cardioneuroablation, while pacemakers should be reserved for older patients. Additionally, while there was a non-significant trend towards greater blood pressure reductions with age, the progressive decline in heart rate reduction across age groups underscores the evolving autonomic compensation mechanisms with aging. Matching our observed age and sex differences in decompensation patterns, Brignole *et al*.^34^ found younger syncope patients rely on heart rate compensation, while older patients use vascular mechanisms, with women showing greater autonomic sensitivity. These findings may have implications for individualized diagnostic and therapeutic strategies in syncope management, particularly in tailoring interventions based on sex- and age-specific autonomic profiles.

While current ESC guidelines^1^ propose that tilt testing may aid in educating patients to recognize prodromes and practice counter-pressure manoeuvres (Class IIb, Level B), our analysis revealed significant differences and poor agreement (κ = 0.06–0.32) between HUTT-induced and spontaneous symptoms. This discordance suggests that the test environment may not reliably replicate real-world prodromal patterns. Consequently, clinicians should interpret HUTT symptom profiles cautiously when counselling patients, as overreliance on test-specific warnings could undermine injury prevention efforts.

## Limitations

The present study has some limitations. First, the population was drawn from a tertiary centre and may not be representative of the general population with VVS. For instance, the mean age of our population and the proportion of males were higher than in a typical VVS study, and patients > 80 years were underrepresented. Second, medication discontinuation before HUTT may have influenced symptom agreement and test positivity, especially in case of iatrogenic low BP, though this protocol was necessary for standardized testing and identifying accurate types of response. Third, we only included patients who had undergone HUTT, which may introduce selection bias and limit the generalizability of our findings. Fourth, we could not assess some common triggers (prolonged standing, micturition) as they were not systematically captured in our questionnaires. Finally, we lack access to implantable loop recorders, and when available, they are prohibitively expensive and not covered by insurance, making them inaccessible for most patients. For recurrent cases, we rely on one-week Holter monitoring.

## Conclusion

This study highlights significant sex- and age-related differences in the clinical presentation, prodromal symptoms, and haemodynamic responses in patients with VVS. These differences provide valuable insights into the pathophysiology of VVS and suggest that a tailored diagnostic and therapeutic approach, considering both sex and age, could improve patient management. Females, particularly younger patients, exhibit more specific triggers and prodromal symptoms, while older patients tend to have more subtle presentations and fewer identifiable warning signs. Given these findings, further studies with larger and more diverse patient populations are necessary to refine our understanding of vasovagal syncope and to develop personalized treatment strategies that address the distinct needs of different patient groups.

## Supplementary Material

oeaf061_Supplementary_Data

## Data Availability

Data are available from the corresponding author upon reasonable request.

## Lead author biography

Dr Masih Tajdini is a board-certified cardiologist from Tehran University of Medical Sciences and an NIH-funded research fellow at Johns Hopkins University. Formerly an Associate Professor, he established the syncope clinic and database at Tehran Heart Center and led numerous single- and multicentre clinical trials on vasovagal syncope. He was nominated for the ESC Young Investigator Award in 2020 and the W. Leigh Thompson Excellence in Clinical Research Award at Hopkins in 2025. His research focuses on autonomic dysfunction and vasovagal syncope, with ongoing contributions to top cardiology journals and international conferences.

## References

[1] Brignole M, Moya A, De Lange FJ, Deharo JC, Elliott PM, Fanciulli A, Fedorowski A, Furlan R, Kenny RA, Martín A, Probst V, Reed MJ, Rice CP, Sutton R, Ungar A, van Dijk JG, Torbicki A, Moreno J, Aboyans V, Agewall S, Asteggiano R, Blanc J-J, Bornstein N, Boveda S, Bueno H, Burri H, Coca A, Collet J-P, Costantino G, Díaz-Infante E, Delgado V, Dolmans F, Gaemperli O, Gajek J, Hindricks G, Kautzner J, Knuuti J, Kulakowski P, Lambrinou E, Leclercq C, Mabo P, Morillo CA, Piepoli MF, Roffi M, Shen WK, Simpson IA, Stockburger M, Vanbrabant P, Windecker S, Zamorano JL, Windecker S, Aboyans V, Agewall S, Barbato E, Bueno H, Coca A, Collet J-P, Coman IM, Dean V, Delgado V, Fitzsimons D, Gaemperli O, Hindricks G, Iung B, Jüni P, Katus HA, Knuuti J, Lancellotti P, Leclercq C, McDonagh T, Piepoli MF, Ponikowski P, Richter DJ, Roffi M, Shlyakhto E, Sousa-Uva M, Simpson IA, Zamorano JL, Roithinger FX, Chasnoits A, Vandekerckhove Y, Traykov VB, Puljevic D, Papasavvas E, Kautzner J, Mølgaard H, Nawar M, Parikka H, Vavlukis M, Piot O, Etsadashvili K, Klingenheben T, Deftereos S, Sághy L, Gudmundsson K, Beinart R, Raviele A, Abdrakhmanov A, Mirrakhimov E, Kalejs O, Benlamin HA, Puodziukynas A, Dimmer C, Sammut MA, Raducan A, Vukmirović M, Abdelali S, Hemels MEW, Haugaa KH, Baranowski R, Cunha PS, Dan G-A, Tyurina T, Bertelli L, Mitro P, Lozano IF, Bergfeldt L, Osswald S, Afef BH, Özdemír HM, Lim PB. 2018 ESC Guidelines for the diagnosis and management of syncope. Eur Heart J 2018;39:1883–1948. 29562304 10.1093/eurheartj/ehy037

[2] Ganzeboom KS, Mairuhu G, Reitsma JB, Linzer M, Wieling W, van Dijk N. Lifetime cumulative incidence of syncope in the general population: a study of 549 Dutch subjects aged 35–60 years. J Cardiovasc Electrophysiol 2006;17:1172–1176. 17074006 10.1111/j.1540-8167.2006.00595.x

[3] Wieling W, Jardine DL, De Lange FJ, Brignole M, Nielsen HB, Stewart J, Sutton R. Cardiac output and vasodilation in the vasovagal response: an analysis of the classic papers. Heart Rhythm 2016;13:798–805. 26598322 10.1016/j.hrthm.2015.11.023 PMC5234327

[4] van Dijk JG, Wieling W. Pathophysiological basis of syncope and neurological conditions that mimic syncope. Prog Cardiovasc Dis 2013;55:345–356. 23472770 10.1016/j.pcad.2012.10.016

[5] Romme JJCM, Van Dijk N, Boer KR, Dekker LRC, Stam J, Reitsma JB, Wieling W. Influence of age and gender on the occurrence and presentation of reflex syncope. Clin Auton Res 2008;18:127–133. 18449594 10.1007/s10286-008-0465-0

[6] Rivasi G, Torabi P, Secco G, Ungar A, Sutton R, Brignole M, Fedorowski A. Age-related tilt test responses in patients with suspected reflex syncope. EP Europace 2021;23:1100–1105. 33564843 10.1093/europace/euab024

[7] Zimmermann T, du Fay de Lavallaz J, Nestelberger T, Gualandro DM, Strebel I, Badertscher P, Lopez-Ayala P, Widmer V, Freese M, Miró Ò, Christ M, Cullen L, Than M, Martin-Sanchez FJ, Di Somma S, Peacock WF, Keller DI, Boeddinghaus J, Twerenbold R, Wussler D, Koechlin L, Walter JE, Bürgler F, Geigy N, Kühne M, Reichlin T, Lohrmann J, Mueller C. Incidence, characteristics, determinants, and prognostic impact of recurrent syncope. Europace 2020;22:1885–1895. 33038231 10.1093/europace/euaa227

[8] Bernier R, Tran DT, Sheldon RS, Kaul P, Sandhu RK. A population-based study evaluating sex differences in patients presenting to emergency departments with syncope. JACC Clin Electrophysiol 2020;6:341–347. 32192686 10.1016/j.jacep.2019.11.002

[9] van Dijk JG, van Rossum IA, van Houwelingen M, Ghariq M, Saal DP, de Lange FJ, Thijs RD, Sutton R, Benditt DG. Influence of age on magnitude and timing of vasodepression and cardioinhibition in tilt-induced vasovagal syncope. JACC Clin Electrophysiol 2022;8:997–1009. 35981805 10.1016/j.jacep.2022.05.009

[10] Deveau AP, Sheldon R, Maxey C, Ritchie D, Doucette S, Parkash R. Sex differences in vasovagal syncope: a post hoc analysis of the prevention of syncope trials (POST) I and II. Can J Cardiol 2020;36:79–83. 31810744 10.1016/j.cjca.2019.10.008

[11] Raj SR, Ahmed SB, Sheldon RS. Understanding vasovagal syncope: a role for sex and gender. Clin Auton Res 2020;30:369–370. 32300948 10.1007/s10286-020-00689-y PMC7572487

[12] Tajdini M, Tavolinejad H, Aminorroaya A, Aryan Z, Jalali A, Alaeddini F, Sadeghian S, Yadangi S, Vasheghani-Farahani A, Kalhor P, Bozorgi A. Clinical associations of injuries caused by vasovagal syncope: a cohort study from a tertiary syncope unit. J Am Heart Assoc 2023;12:e027272. 36565190 10.1161/JAHA.122.027272 PMC9973565

[13] Fedorowski A, Kulakowski P, Brignole M, De Lange FJ, Kenny RA, Moya A, Rivasi G, Sheldon R, Van Dijk G, Sutton R. Twenty-five years of research on syncope. Europace 2023;25:1–13. 37622579 10.1093/europace/euad163 PMC10450792

[14] Groppelli A, Russo V, Parente E, Comune A, De Lange FJ, Rivasi G, Rivasi G, Rafanelli M, Deharo JC, Francisco-Pascual J, Maggi R, Fedorowski A, Ungar A, Parati G, Brignole M. Mechanism of syncope: role of ambulatory blood pressure monitoring and cardiovascular autonomic function assessment. Eur Heart J 2025;46:827–835. 39786439 10.1093/eurheartj/ehae907 PMC11879164

[15] Shen W-K, Sheldon RS, Benditt DG, Cohen MI, Forman DE, Goldberger ZD, Grubb BP, Hamdan MH, Krahn AD, Link MS. 2017 ACC/AHA/HRS guideline for the evaluation and management of patients with syncope: executive summary: a report of the American College of Cardiology/American Heart Association Task Force on Clinical Practice Guidelines and the Heart Rhythm Society. Circulation 2017;136:e60–e122. 28280231 10.1161/CIR.0000000000000499

[16] Bartoletti A, Alboni P, Ammirati F, Brignole M, Del Rosso A, Foglia Manzillo G, Menozzi C, Raviele A, Sutton R. “The Italian Protocol”: a simplified head-up tilt testing potentiated with oral nitroglycerin to assess patients with unexplained syncope. Europace 2000;2:339–342. 11194602 10.1053/eupc.2000.0125

[17] Parry SW, Reeve P, Lawson J, Shaw FE, Davison J, Norton M, Frearson R, Kerr S, Newton JL. The Newcastle protocols 2008: an update on head-up tilt table testing and the management of vasovagal syncope and related disorders. Heart 2008;95:416–420. 18701533 10.1136/hrt.2007.136457

[18] Torabi P, Hamrefors V, Sutton R, Brignole M, Fedorowski A. Definitive aetiology of unexplained syncope after cardiovascular autonomic tests in a tertiary syncope unit. Europace 2023;25:1–8. 37589189 10.1093/europace/euad247 PMC10505743

[19] Fedorowski A, Pirouzifard M, Sundquist J, Sundquist K, Sutton R, Zöller B. Risk factors for syncope associated with multigenerational relatives with a history of syncope. JAMA Netw Open 2021;4:e212521. 33783519 10.1001/jamanetworkopen.2021.2521 PMC8010588

[20] van Dijk N, Sprangers MA, Boer KR, Colman N, Wieling W, Linzer M. Quality of life within one year following presentation after transient loss of consciousness. Am J Cardiol 2007;100:672–676. 17697827 10.1016/j.amjcard.2007.03.085

[21] Jardine DL, Wieling W, Brignole M, Lenders JWM, Sutton R, Stewart J. The pathophysiology of the vasovagal response. Heart Rhythm 2018;15:921–929. 29246828 10.1016/j.hrthm.2017.12.013 PMC5984661

[22] Muppa P, Sheldon RS, McRae M, Keller NR, Ritchie D, Krahn AD, Morillo CA, Kus T, Talajic M, Raj SR. Gynecological and menstrual disorders in women with vasovagal syncope. Clin Auton Res 2013;23:117–122. 23467969 10.1007/s10286-013-0190-1 PMC3681885

[23] Wenner MM, Stachenfeld NS. Blood pressure and water regulation: understanding sex hormone effects within and between men and women. J Physiol 2012;590:5949. 23027816 10.1113/jphysiol.2012.236752 PMC3530109

[24] Jarjour IT, Jarjour LK. Low iron storage in children and adolescents with neurally mediated syncope. J Pediatr 2008;153:40–44.e1. 18571533 10.1016/j.jpeds.2008.01.034

[25] Raj SR, Coffin ST. Medical therapy and physical maneuvers in the treatment of the vasovagal syncope and orthostatic hypotension. Prog Cardiovasc Dis 2013;55:425–433. 23472781 10.1016/j.pcad.2012.11.004 PMC3594734

[26] Moya A, Brignole M, Menozzi C, Garcia-Civera R, Tognarini S, Mont L, Botto G, Giada F, Cornacchia D. Mechanism of syncope in patients with isolated syncope and in patients with tilt-positive syncope. Circulation 2001;104:1261–1267. 11551877 10.1161/hc3601.095708

[27] Joza J, Gustavo Bravosi da Rosa L, Alturki A, Anglesio V, Sanchez-Somonte P, Poletaev V, Bernier M, Verma A, Essebag V. Cardioneuroablation as a strategy to prevent pacemaker implantation in young patients with vasovagal syncope. Int J Cardiol Heart Vasc 2024;51:101360. 38379634 10.1016/j.ijcha.2024.101360 PMC10877404

[28] Aksu T, Brignole M, Calo L, Debruyne P, Di Biase L, Deharo JC, Fanciulli A, Fedorowski A, Kulakowski P, Morillo C, Moya A, Piotrowski R, Stec S, Sutton R, van Dijk JG, Wichterle D, Tse H-F, Yao Y, Sheldon RS, Vaseghi M, Pachon JC, Scanavacca M, Meyer C, Amin R, Gupta D, Magnano M, Malik V, Schauerte P, Shen W-K, Acosta JCZ. Cardioneuroablation for the treatment of reflex syncope and functional bradyarrhythmias: a scientific statement of the European Heart Rhythm Association (EHRA) of the ESC, the Heart Rhythm Society (HRS), the Asia Pacific Heart Rhythm Society (APHRS) and the Latin American Heart Rhythm Society (LAHRS). Europace 2024;26. doi:10.1093/europace/euae206. PMC11350289 39082698

[29] Asad M, Khan K, Nazir MT, Ali M, Nazir A, Khan HS, Khan A, Khan S, Khan QH. Age and gender related outcomes in patients undergoing head up tilt test for vasovagal syncope. PAFMJ 2021;71:S368–S371.

[30] Fluckiger L, Boivin J-M, Quilliot D, Jeandel C, Zannad F. Differential effects of aging on heart rate variability and blood pressure variability. J Gerontol A Biol Sci Med Sci 1999;54:B219–B224. 10362001 10.1093/gerona/54.5.b219

[31] Russo V, Parente E, Rago A, Comune A, Laezza N, Papa AA, Chamberland C, Huynh T, Golino P, Brignole M, Nigro G. Cardioinhibitory syncope with asystole during nitroglycerin potentiated head up tilt test: prevalence and clinical predictors. Clin Auton Res 2022;32:167–173. 35524080 10.1007/s10286-022-00864-3 PMC9236999

[32] Xu L, Cao X, Wang R, Duan Y, Yang Y, Hou J, Wang J, Chen B, Xue X, Zhang B, Ma H, Sun C, Guo F. Clinical features of patients undergoing the head-up tilt test and its safety and efficacy in diagnosing vasovagal syncope in 4,873 patients. Front Cardiovasc Med 2021;8:781157. 35097001 10.3389/fcvm.2021.781157 PMC8790085

[33] Baker SE, Limberg JK, Dillon GA, Curry TB, Joyner MJ, Nicholson WT. Aging alters the relative contributions of the sympathetic and parasympathetic nervous system to blood pressure control in women. Hypertension 2018;72:1236–1242. 30354803 10.1161/HYPERTENSIONAHA.118.11550 PMC6211807

[34] Brignole M, Rivasi G, Sutton R, Kenny RA, Morillo CA, Sheldon R, Raj SR, Ungar A, Furlan R, van Dijk G, Hamdan M, Hamrefors V, Engström G, Park C, Soranna D, Zambon A, Parati G, Fedorowski A. Low-blood pressure phenotype underpins the tendency to reflex syncope. J Hypertens 2021;39:1319–1325. doi:10.1097/HJH.0000000000002800. 33560050 PMC8183486

