# Correction to: Age and sex differences in vasovagal syncope: triggers, clinical presentation, prodromal symptoms, and head-up tilt test results

**DOI:** 10.1093/ehjopen/oeaf079

**Published:** 2025-06-23

This is a correction to: Mohammadreza Babaei, Masih Tajdini, Ali Bozorgi, Saeed Sadeghian, Morvarid Taebi, Hamed Tavolinejad, Mehrdad Mahalleh, Homa Taheri, Florian Rader, Jeffrey R Boris, Artur Fedorowski, Age and sex differences in vasovagal syncope: triggers, clinical presentation, prodromal symptoms, and head-up tilt test results, *European Heart Journal Open*, Volume 5, Issue 3, May 2025, https://doi.org/10.1093/ehjopen/oeaf061

The following changes have been made to the originally published manuscript. The transposed names of the ninth co-author have been emended to read: “Florian Rader”. The contact for the corresponding author has been emended to read: “P.O. Box 16 Moylan, PA 19065, USA” instead of: “27 East Greenwood Avenue, Moylan, PA 19065, USA”.

