# Supplementary material for: Age and sex differences in vasovagal syncope: triggers, clinical presentation, prodromal symptoms, and head-up tilt test results
Source: Eur Heart J Open. 2025 Jun 11;5(3):oeaf061. doi: 10.1093/ehjopen/oeaf061 (PMC12152479; doi:10.1093/ehjopen/oeaf061)
Supplement: oeaf061_Supplementary_Data [file oeaf061_supplementary_data.pdf]

Supplementary Material

Age and Sex Differences in Vasovagal Syncope: Triggers, Clinical presentation, Prodromal symptoms, and Head-up tilt test results

This supplemental material has been provided by the authors to present readers with additional information about their work.

Table of Contents

Supplementary Figure 1. Age distribution across decadal groups 3
Supplementary Figure 2. Type of HUTT response across decadal groups 4
Supplementary Figure 3. Hemodynamic change in patients with negative tilt test 5
Supplementary Table 1. Triggers in HUTT-positive patients based on sex and age groups....6
Supplementary Table 2. Prodromal symptoms of spontaneous syncope based on sex and age group....7
Supplementary Table 3. Presenting symptoms of VVS in the total population based on sex....8
Supplementary Table 4. Symptoms after spontaneous syncope based on sex and age 9
Supplementary Table 5. Characteristics and differences of HUTT-positive patients based on sex 10
Supplementary Table 6. HUTT findings among patients with positive tilt results based on sex and age group 11
Supplementary Table 7. Prodromal symptoms of HUTT-induced syncope in HUTT-positive patients based on sex and age group....12
Supplementary Table 8. Agreement between prodromal symptoms of spontaneous syncope and HUT-induced syncope based on sex....13

Supplementary Figure 1. Age distribution across decadal groups

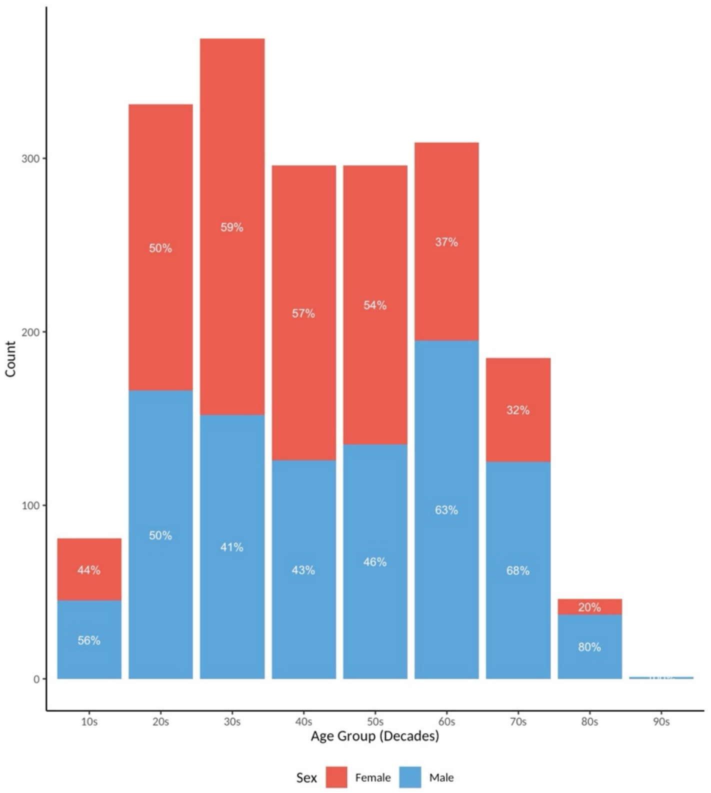

Data are presnted as percentages.

Supplementary Figure 2. Type of HUTT response across decadal groups

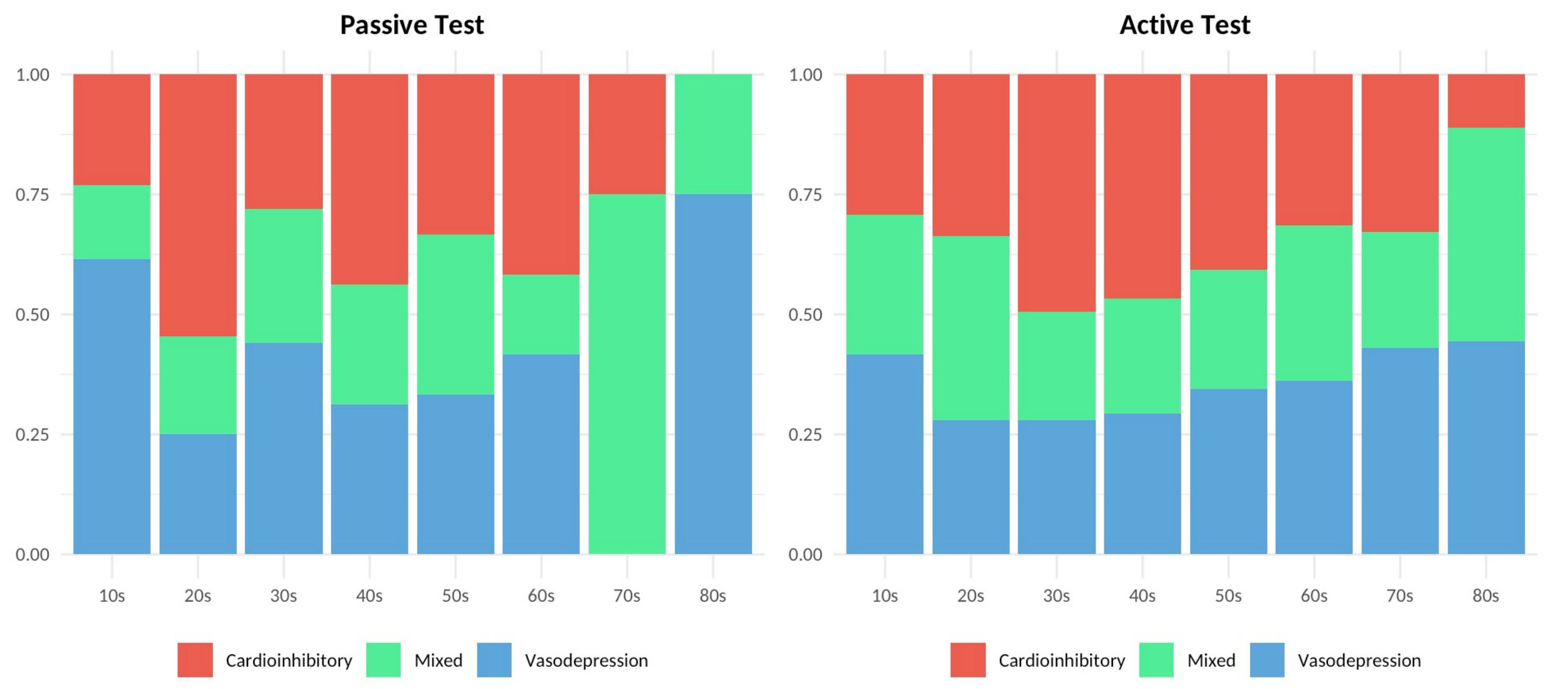

Data are presnted as percentages. HUTT= head-up tilt test

Supplementary Figure 3. Hemodynamic change in patients with negative tilt test

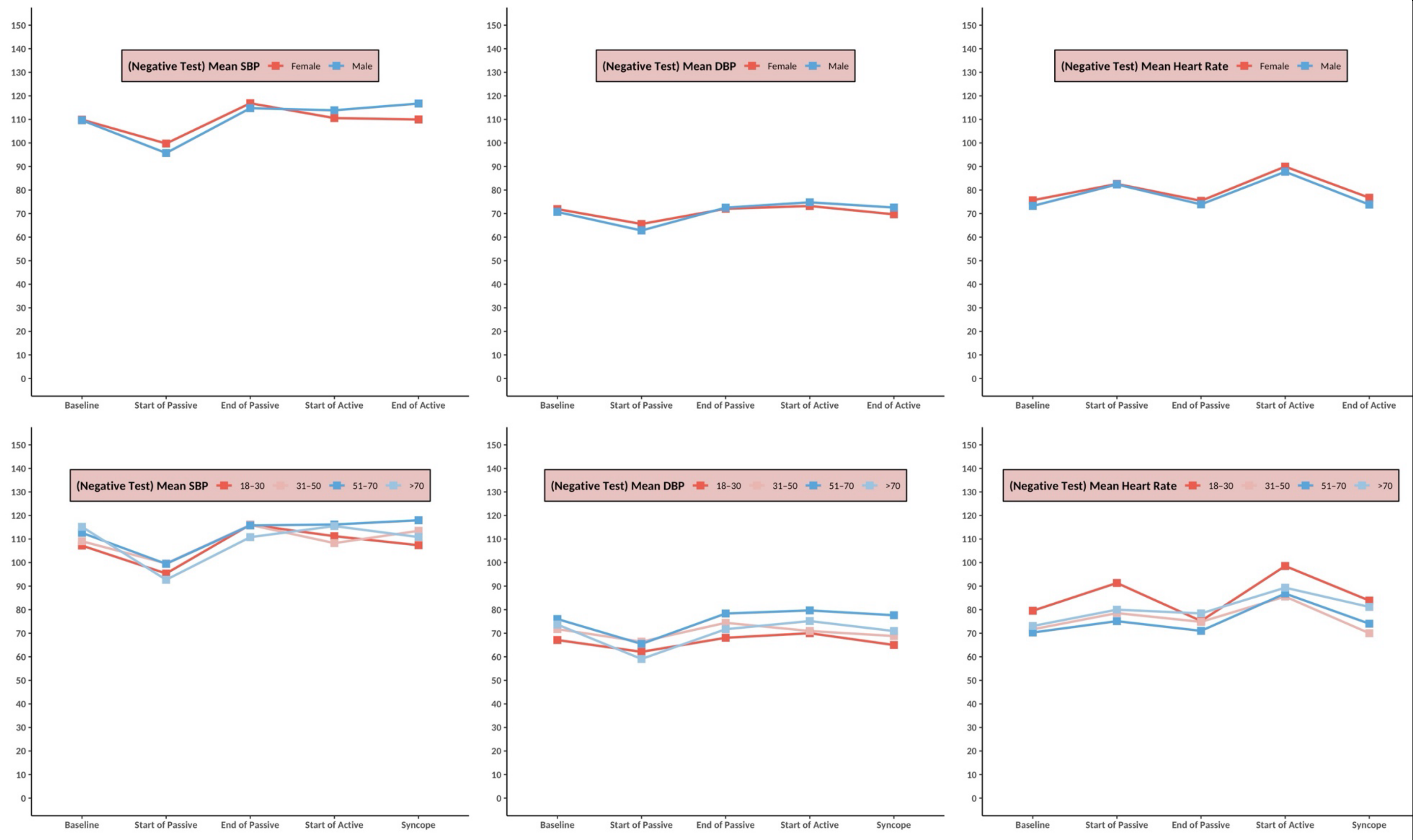

SBP = Systolic blood pressure, DBP = Diastolic blood pressure

Supplementary Table 1. Triggers in HUTT-positive patients based on sex and age groups

HUTT Positive	Overal	*Sex group*			*Age group (years)*				
	**Overal** (N = 764)	**Female** (n = 383)	**Male** (n = 381)	***P*–value**	**18–30** (n = 446)	**31–50** (n = 660)	**51–70** (n = 601)	**>70** (n = 207)	***P*–value**
Crowded or warm environment	133 (17.4)	82 (21.4)	51 (13.4)	0.005	55 (26.8)	44 (17.7)	31 (13.8)	3 (3.5)	<0.001
Exposure to hot water	106 (13.9)	59 (15.4)	47 (12.3)	0.262	43 (21.0)	38 (15.3)	21 (9.3)	4 (4.7)	<0.001
Sight of blood	91 (11.9)	37 (9.7)	54 (14.2)	0.070	42 (20.5)	29 (11.7)	17 (7.6)	3 (3.5)	<0.001
Emotional stress	79 (10.3)	44 (11.5)	35 (9.2)	0.354	25 (12.2	30 (12.1)	21 (9.3)	3 (3.5)	0.103
Pain	72 (9.4)	44 (11.5)	28 (7.3)	0.067	32 (15.6)	28 (11.3)	11 (4.9)	1 (1.2)	<0.001
Medical or dental procedure	45 (5.9)	31 (8.1)	14 (3.7)	0.015	10 (4.9)	20 (8.1)	15 (6.7)	0 (0.0)	0.044

Data are presented as numbers (percentages).

Supplementary Table 2. Prodromal symptoms of spontaneous syncope based on sex and age group

**Total Population**		*Sex group*			*Age group*				
	**Overall** (n = 1914)	**Female (**n = 932)	**Male** (n = 982)	***P*–value**	**18–30** (n = 446)	**31–50** (n = 660)	**51–70** (n = 601)	**>70 (**n = 207)	***P*–value**
**Presence of any prodromes** (%)	1575 (82.3)	785 (84.2)	790 (80.4)	0.035	405 (90.8)	563 (85.3)	469 (78.0)	138 (66.7)	<0.001
Lightheadedness	1072 (56.0)	529 (56.8)	543 (55.3)	0.549	302 (67.7)	378 (57.3)	294 (48.9)	98 (47.3)	<0.001
Blurred vision	922 (48.2)	451 (48.4)	471 (48.0)	0.888	275 (61.7)	342 (51.8)	237 (39.4)	68 (32.9)	<0.001
Diaphoresis	520 (27.2)	259 (27.8)	261 (26.6)	0.586	116 (26.0)	210 (31.8)	162 (27.0)	32 (15.5)	<0.001
Nausea	487 (25.4)	279 (29.9)	208 (21.2)	<0.001	133 (29.8)	203 (30.8)	119 (19.8)	32 (15.5)	<0.001
Palpitation	523 (27.3)	319 (34.2)	204 (20.8)	<0.001	171 (38.3)	208 (31.5)	129 (21.5)	15 (7.2)	<0.001
Heat feeling	404 (21.1)	227 (24.4)	177 (18.0)	0.001	124 (27.8)	151 (22.9)	112 (18.6)	17 (8.2)	<0.001
Chest Pain	240 (12.5)	144 (15.5)	96 (9.8)	<0.001	66 (14.8)	101 (15.3)	61 (10.1)	12 (5.8)	<0.001
Abdominal discomfort	151 (7.9)	104 (11.2)	47 (4.8)	<0.001	48 (10.8)	58 (8.8)	39 (6.5)	6 (2.9)	0.002
Flushing	145 (7.6)	86 (9.2)	59 (6.0)	0.010	34 (7.6)	70 (10.6)	37 (6.2)	4 (1.9)	<0.001
Aura	80 (4.2)	52 (5.6)	28 (2.9)	0.004	28 (6.3)	33 (5.0)	17 (2.8)	2 (1.0)	0.003
**HUTT Positive**		*Sex group*			*Age group*				
	**Overall** (n = 764)	**Female (**n = 383)	**Male** (n = 381)	***P*–value**	**18–30** (n = 205)	**31–50** (n = 248)	**51–70** (n = 225)	**>70 (**n = 86)	***P*–value**
**Presence of prodromes** (%)	641 (83.9)	325 (84.9)	316 (82.9)	0.534	193 (94.1)	205 (82.7)	188 (83.6)	55 (64)	<0.001
Lightheadedness	419 (54.8)	210 (54.8)	209 (54.9)	>0.999	134 (65.4)	138 (55.6)	110 (48.9)	37 (43.0)	0.001
Blurred vision	380 (49.7)	179 (46.7)	201 (52.8)	0.111	128 (62.4)	129 (52.0)	99 (44.0)	24 (27.9)	<0.001
Diaphoresis	212 (27.7)	104 (27.2)	108 (28.3)	0.774	52 (25.4)	74 (29.8)	71 (31.6)	15 (17.4)	0.063
Nausea	206 (27.0)	121 (31.6)	85 (22.3)	0.005	61 (29.8)	77 (31.0)	49 (21.8)	19 (22.1)	0.071
Palpitation	187 (24.5)	113 (29.5)	74 (19.4)	0.002	69 (33.7)	73 (29.4)	41 (18.2)	4 (4.7)	<0.001
Heat feeling	175 (22.9)	96 (25.1)	79 (20.7)	0.181	59 (28.8)	58 (23.4)	52 (23.1)	6 (7.0)	0.001
Chest Pain	83 (10.9)	48 (12.5)	35 (9.2)	0.171	23 (11.2)	35 (14.1)	20 (8.9)	5 (5.8)	0.117
Abdominal discomfort	74 (9.7)	52 (13.6)	22 (5.8)	<0.001	23 (11.2)	29 (11.7)	21 (9.3)	1 (1.2)	0.031
Flushing	63 (8.2)	32 (8.4)	31 (8.1)	>0.999	15 (7.3)	31 (12.5)	16 (7.1)	1 (1.2)	0.007
Aura	31 (4.1)	23 (6.0)	8 (2.1)	0.011	11 (5.4)	14 (5.6)	5 (2.2)	1 (1.2)	0.098

Categorical variables are shown as numbers (percentage). GI= Gastrointestinal;

Supplementary Table 3. Presenting symptoms of VVS in the total population based on sex

		Sex group			***Age group (years)***				
**Total Population**	**Overall** (N = 1914)	**Female** (n = 932)	**Male** (n = 982)	***P*–value**	**18–30 (n = 446)**	**31–50 (n = 660)**	**51–70 (n = 601)**	**>70 (n = 207)**	***P*–value**
Seizure-like movements	173 (9.0)	101 (10.8)	72 (7.3)	0.010	66 (14.8)	77 (11.7)	25 (4.2)	5 (2.4)	<0.001
Tongue biting	87 (4.5)	47 (5.0)	40 (4.1)	0.364	25 (5.6)	41 (6.2)	18 (3.0)	3 (1.4)	0.004
Abnormal gaze	145 (7.6)	79 (8.5)	66 (6.7)	0.172	54 (12.1)	53 (8.0)	30 (5.0)	8 (3.9)	<0.001
Urinary incontinence	124 (6.5)	67 (7.2)	57 (5.8)	0.256	21 (4.7)	40 (6.1)	44 (7.3)	19 (9.2)	0.128
		Sex group			***Age group (years)***				
**HUTT Positive**	**Overall** (n = 764)	**Female** (n = 383)	**Male** (n = 381)	***P*–value**	**18–30** (n = 446)	**31–50** (n = 660)	**51–70** (n = 601)	**>70** (n = 207)	***P*–value**
Seizure-like movements	80 (10.5)	48 (12.5)	32 (8.4)	0.081	34 (16.6)	32 (12.9)	9 (4.0)	5 (5.8)	<0.001
Tongue biting	29 (3.8)	17 (4.4)	12 (3.1)	0.458	7 (3.4)	17 (6.9)	3 (1.3)	2 (2.3)	0.014
Abnormal gaze	77 (10.1)	45 (11.7)	32 (8.4)	0.156	26 (12.7)	27 (10.9)	19 (8.4)	5 (5.8)	0.251
Urinary incontinence	53 (6.9)	33 (8.6)	20 (5.2)	0.091	10 (4.9)	21 (8.5)	14 (6.2)	8 (9.3)	0.366

Data are presented in numbers (percentages).

Supplementary Table 4. Symptoms after spontaneous syncope based on sex and age

Total Population		*Sex group*			*Age group (years)*				
	**Overall** (N = 1914)	**Female** (n = 932)	**Male** (n = 982)	***P*–value**	**18–30** (N = 446)	**31–50** (N = 660)	**51–70** (N = 601)	**>70** (N = 207)	***P*–value**
Drowsiness	531 (27.7)	314 (33.7)	217 (22.1)	<0.001	147 (33.0)	225 (34.1)	129 (21.5)	30 (14.5)	<0.001
Headache	496 (25.9)	285 (30.6)	211 (21.5)	<0.001	173 (38.8)	210 (31.8)	93 (15.5)	20 (9.7)	<0.001
Palpitation	356 (18.6)	212 (22.7)	144 (14.7)	<0.001	122 (27.4)	143 (21.7)	81 (13.5)	10 (4.8)	<0.001
Retrograde amnesia	265 (13.8)	150 (16.1)	115 (11.7)	0.006	75 (16.8)	107 (16.2)	62 (10.3)	21 (10.1)	0.002
Chest discomfort	213 (11.1)	125 (13.4)	88 (9.0)	0.002	53 (11.9)	99 (15.0)	54 (9.0)	7 (3.4)	<0.001

HUTT Positive		*Sex group*			*Age group (years)*				
	**Overall** (N = 1914)	**Female** (n = 932)	**Male** (n = 982)	***P*–value**	**18–30** **(n = 446)**	**31–50** **(n = 660)**	**51–70** **(n = 601)**	**>70** **(n = 207)**	***P*–value**
Drowsiness	207 (27.1)	122 (31.9)	85 (22.3)	0.004	147 (33.0)	225 (34.1)	129 (21.5)	30 (14.5)	<0.001
Headache	194 (25.4)	109 (28.5)	85 (22.3)	0.062	173 (38.8)	210 (31.8)	93 (15.5)	20 (9.7)	<0.001
Palpitation	144 (18.8)	86 (22.5)	58 (15.2)	0.014	122 (27.4)	143 (21.7)	81 (13.5)	10 (4.8)	<0.001
Retrograde amnesia	106 (13.9)	58 (15.1)	48 (12.6)	0.361	75 (16.8)	107 (16.2)	62 (10.3)	21 (10.1)	0.002
Chest discomfort	75 (9.8)	46 (12.0)	29 (7.6)	0.055	53 (11.9)	99 (15.0)	54 (9.0)	7 (3.4)	<0.001

Data are presented as numbers (percentages).

Supplementary Table 5. Characteristics and differences of HUTT-positive patients based on sex

		Sex group			*Age group (year)*				
	**Overal (n = 764)**	**Female (n = 383)**	**Male (n = 381)**	***P*–value**	**18–30 (n = 205)**	**31–50 (n = 248)**	**51–70 (n = 225)**	**>70 (n = 86)**	***P* –value**
**Age**, year	45.8 ± 18.4	43.1 ± 16.7	48.5 ± 19.7	<0.001	–	–	–	–	–
**Female**	–	–	–	–	106 (51.7)	149 (60.1)	103 (45.8)	25 (29.1)	<0.001
**BMI**	26.2 ± 14.3	25.8 ± 5.1	26.6 ± 19.6	0.480	22.9 ± 4.6	26.4 ± 4.6	29.4 ± 5.2	25.1 ± 3.4	<0.001
**Diabetes mellitus**	53 (6.9)	15 (3.9)	38 (10.0)	0.002	3 (1.5)	7 (2.8)	28 (12.4)	15 (17.4)	<0.001
**Hypertension**	152 (19.9)	59 (15.4)	93 (24.4)	0.002	8 (3.9)	32 (12.9)	72 (32.0)	40 (46.5)	<0.001
**Anemia**	140 (18.3)	89 (23.2)	51 (13.4)	0.001	38 (18.5)	50 (20.2)	37 (16.4)	15 (17.4)	0.767
**Last-year episodes**				0.430					0.004
1	331 (48.5)	159 (46.8)	172 (50.3)		75 (39.9)	119 (52.2)	103 (53.9)	34 (45.3)	
2	135 (19.8)	68 (20.0)	67 (19.6)		37 (19.7)	39 (17.1)	38 (19.9)	21 (28.0)	
3	64 (9.4)	29 (8.5)	35 (10.2)		17 (9.0)	17 (7.5)	20 (10.5)	10 (13.3)	
≥4	152 (22.3)	84 (24.7)	68 (19.9)		59 (31.4)	53 (23.2)	30 (15.7)	10 (13.3)	
**Lifetime episodes**				0.002					0.001
1	152 (22.3)	84 (24.7)	68 (19.9)		35 (18.6)	59 (25.9)	57 (29.8)	24 (32.4)	
2	135 (19.8)	76 (22.4)	59 (17.3)		29 (15.4)	45 (19.7)	41 (21.5)	20 (27.0)	
3	86 (12.6)	37 (10.9)	49 (14.4)		20 (10.6)	29 (12.7)	25 (13.1)	12 (16.2)	
≥4	285 (41.9)	158 (46.5)	127 (37.2)		104 (55.3)	95 (41.7)	68 (35.6)	18 (24.3)	
**History of syncope associated injury** (%)	131 (17.1)	76 (19.8)	55 (14.1)	0.059	38 (18.5)	43 (17.3)	34 (15.1)	16 (18.6)	0.784

Categorical variables are shown as numbers (percentage) and continuous variables as mean ± standard deviation.
BMI= body mass index;

**Supplementary Table 6. HUTT findings among patients with positive tilt results based on sex and age group**									
		Sex group			*Age group (year)*				
**HUTT Positive**	**Overall (n = 764)**	**Female (n = 383)**	**Male (n = 381)**	***P*–value**	**18–30** (n = 205)	**31–50** (n = 248)	**51–70** (n = 225)	**>70** (n = 86)	***P*–value**
**Passive** (%)	133 (18.8)	56	77	–	n = 59	41	25	8	–
**Mixed**	33 (24.8)	13 (23.2)	20 (26.0)	0.872	11 (18.6)	11 (26.8)	7 (28.0)	4 (50.0)	0.246
**Cardioinhibitory**	52 (39.1)	23 (41.1)	29 (37.7)	0.828	28 (47.5)	15 (36.6)	8 (32.0)	1 (12.5)	0.191
**Vasodepressor**	48 (36.1)	20 (35.7)	28 (36.4)	1.000	20 (33.9)	15 (36.6)	10 (40.0)	3 (37.5)	0.960
**Mean SBP Reduction**	64.3 ± 22.4	70.9 ± 25.6	59.2 ± 18.6	0.132	60.5 ± 25.3	67.2 ± 24.6	67.7 ± 9.6	86.01	0.664
**Mean DBP Reduction**	40.7 ± 16.8	49.5 ± 12.2	34.4 ± 17.2	0.012	38.7 ± 17.6	38.5 ± 15.0	48.4 ± 18.6	55.0	0.565
**Mean HR Reduction**	28.7 ± 28.0	34.8 ± 23.0	25.0 ± 30.3	0.154	27.9 ± 33.4	29.7 ± 26.8	30.7 ± 15.1	23.2 ± 13.7	0.968
**Active** (%)	573 (81.2)	300	273	–	138	185	181	69	–
**Mixed**	166 (29.0)	90 (30.0)	76 (27.8)	0.633	53 (38.4)	40 (21.6)	52 (28.7)	21 (30.4)	0.012
**Cardioinhibitory**	217 (37.9)	121 (40.3)	96 (35.2)	0.235	42 (30.4)	93 (50.3)	63 (34.8)	19 (27.5)	<0.001
**Vasodepressor**	190 (33.2)	89 (29.7)	101 (37.0)	0.076	43 (31.2)	52 (28.1)	66 (36.5)	29 (42.0)	0.021
**Mean SBP Reduction**	60.2 ± 21.0	60.4 ± 22.1	59.9 ± 19.6	0.840	57.1 ± 21.6	62.6 ± 19.4	61.0 ± 20.6	57.8 ± 24.5	0.378
**Mean DBP Reduction**	41.3 ± 16.5	40.7 ± 16.4	42.1 ± 16.6	0.502	39.9 ± 18.1	43.6 ± 13.8	41.1 ± 16.8	38.3 ± 19.3	0.436
**Mean HR Reduction**	35.0 ± 25.6	39.7 ± 26.9	30.2 ± 23.3	<0.001	42.6 ± 26.3	35.8 ± 27.6	32.0 ± 23.6	28.2 ± 21.9	0.002
Missing values	58 (7.6)	27 (7.0)	31 (8.1)	–	8 (3.9)	22 (8.87)	19 (8.44)	9 (10.47)	–

Head-up tilt test results based on the VASIS-II classification.

Data are presented as numbers (percentages).

**Supplementary Table 7. Prodromal symptoms of HUTT-induced syncope in HUTT-positive patients based on sex and age group**										
**HUTT Positive**		***Sex group***			***Age group (year)***					
	**Overall (n = 764)**	**Female (n = 383)**	**Male (n = 381)**	***P*–value**	**18–30 (n = 205)**	**31–50 (n = 248)**	**51–70 (n = 225)**	**>70 (n = 86)**	***P*–value**
**Lightheadedness**	440 (57.6)	214 (55.9)	226 (59.3)	0.374	128 (62.4)	140 (56.5)	131 (58.2)	41 (47.7)	0.133
**Nausea**	272 (35.6)	142 (37.1)	130 (34.1)	0.437	83 (40.5)	97 (39.1)	68 (30.2)	24 (27.9)	0.036
**Weakness**	254 (33.2)	132 (34.5)	122 (32.0)	0.522	65 (31.7)	82 (33.1)	82 (36.4)	25 (29.1)	0.586
**Diaphoresis**	219 (28.7)	95 (24.8)	124 (32.5)	0.022	47 (22.9)	67 (27.0)	80 (35.6)	25 (29.1)	0.031
**Heat feeling**	147 (19.2)	68 (17.8)	79 (20.7)	0.341	38 (18.5)	42 (16.9)	55 (24.4)	12 (14.0)	0.094
**Palpitation**	112 (14.7)	69 (18.0)	43 (11.3)	0.011	48 (23.4)	43 (17.3)	20 (8.9)	1 (1.2)	<0.001
**Flushing**	101 (13.2)	49 (12.8)	52 (13.6)	0.809	21 (10.2)	34 (13.7)	35 (15.6)	11 (12.8)	0.437
**Gaze**	66 (8.6)	40 (10.4)	26 (6.8)	0.099	21 (10.2)	20 (8.1)	20 (8.9)	5 (5.8)	0.646
**Myoclonus**	59 (7.7)	26 (6.8)	33 (8.7)	0.404	16 (7.8)	21 (8.5)	17 (7.6)	5 (5.8)	0.886
**Chest Pain**	36 (4.7)	18 (4.7)	18 (4.7)	>0.999	11 (5.4)	17 (6.9)	8 (3.6)	0 (0.0)	0.054
**Abdominal discomfort**	31 (4.1)	18 (4.7)	13 (3.4)	0.472	9 (4.4)	11 (4.4)	8 (3.6)	3 (3.5)	0.947
**Aura**	6 (0.8)	6 (1.6)	0 (0.0)	0.041	5 (2.4)	1 (0.4)	0 (0.0)	0 (0.0)	0.018
**Urinary incontinence**	10 (1.3)	7 (1.8)	3 (0.8)	0.344	1 (0.5)	3 (1.2)	3 (1.3)	3 (3.5)	0.235

Categorical variables are shown as numbers (percentage). GI= Gastrointestinal;

Supplementary Table 8. Agreement between prodromal symptoms of spontaneous syncope and HUT-induced syncope based on sex

Symptoms	*Male*					*Female*				
	**McNemar's Chi- test**		**Cohen's kappa**			**McNemar's Chi- test**		**Cohen's kappa**		
	Chi²	***P value***	***Estimates***	***95% CI***	***P value***	***Chi²***	***P value***	***Estimates***	***95% CI***	***P value***
**Diaphoresis**	2.16	0.141	0.36	0.25 – 0.46	<0.001	0.61	0.435	0.29	0.17 – 0.40	<0.001
**Lightheadedness**	1.87	0.171	0.27	0.17 – 0.37	<0.001	18.49	<0.001	0.29	0.17 – 0.41	<0.001
**Palpitation**	11.39	<0.001	0.22	-0.01 – 0.44	0.052	0.06	0.804	0.23	0.13 – 0.33	<0.001
**Nausea**	15.74	<0.001	0.22	0.10 – 0.33	<0.001	2.88	0.089	0.20	0.09 – 0.30	<0.001
**Flushing**	6.56	0.010	0.18	-0.01 – 0.37	0.040	17.52	<0.001	0.22	0.01 – 0.43	0.030
**GI Prodrome**	2.56	0.109	0.25	-0.03 – 0.53	0.062	20.17	<0.001	0.17	-0.03 – 0.38	0.065
**Chest Pain**	6.56	0.010	0.22	-0.02 – 0.45	0.052	12.19	<0.001	0.26	-0.05 – 0.57	0.077
**No symptom**	0.22	0.636	-0.002	-0.16 – 0.15	0.510	3.94	0.047	0.11	-0.09 – 0.31	0.155
**Aura**	–	–	–	–	–	0.52	0.470	0.10	-0.06 – 0.25	0.125

CI= confidence interval; GI= gastrointestinal;

Kappa interpretation, "< 0": No agreement; "0–0.2": Slight agreement; "0.2–0.4": Fair agreement; "0.4–0.6": Moderate agreement; "0.6–0.8": Substantial agreement; "0.8–1.0": Almost perfect agreement;
